# Not All Fibers Are Born Equal; Variable Response to Dietary Fiber Subtypes in IBD

**DOI:** 10.3389/fped.2020.620189

**Published:** 2021-01-15

**Authors:** Heather Armstrong, Inderdeep Mander, Zhengxiao Zhang, David Armstrong, Eytan Wine

**Affiliations:** ^1^Centre of Excellence for Gastrointestinal Inflammation and Immunity Research, University of Alberta, Edmonton, AB, Canada; ^2^Department of Pediatrics, University of Alberta, Edmonton, AB, Canada; ^3^Department of Medicine, University of Alberta, Edmonton, AB, Canada; ^4^Department of Chemical and Physical Sciences, University of Toronto Mississauga, Mississauga, ON, Canada; ^5^Department of Physiology, University of Alberta, Edmonton, AB, Canada

**Keywords:** ulcerative colitis, Crohn disease, IBD–inflammatory bowel diseases, pediatric IBD, dietary fiber

## Abstract

Diet provides a safe and attractive alternative to available treatment options in a variety of diseases; however, research has only just begun to elucidate the role of diet in chronic diseases, such as the inflammatory bowel diseases (IBD). The chronic and highly debilitating IBDs, Crohn disease and ulcerative colitis, are hallmarked by intestinal inflammation, immune dysregulation, and dysbiosis; and evidence supports a role for genetics, microbiota, and the environment, including diet, in disease pathogenesis. This is true especially in children with IBD, where diet-based treatments have shown excellent results. One interesting group of dietary factors that readily links microbiota to gut health is dietary fibers. Fibers are not digested by human cells, but rather fermented by the gut microbes within the bowel. Evidence has been mounting over the last decade in support of the importance of dietary fibers in the maintenance of gut health and in IBD; however, more recent studies highlight the complexity of this interaction and importance of understanding the role of each individual dietary fiber subtype, especially during disease. There are roughly ten subtypes of dietary fibers described to date, categorized as soluble or insoluble, with varying chemical structures, and large differences in their fermentation profiles. Many studies to date have described the benefits of the byproducts of fermentation in healthy individuals and the potential health benefits in select disease models. However, there remains a void in our understanding of how each of these individual fibers affect human health in dysbiotic settings where appropriate fermentation may not be achieved. This review highlights the possibilities for better defining the role of individual dietary fibers for use in regulating inflammation in IBD.

## Introduction

The etiology of the chronic and severely debilitating Inflammatory Bowel Diseases (IBD), Crohn disease (CD), and ulcerative colitis (UC), remains poorly understood and incidence rates are increasing, especially in children (1–3). Risk factors associated with IBD include urban lifestyle, lack of greenspace (4), genetic factors, heightened hygiene, dietary factors, and changes in the microbiome (5–11). Gut microbes are critical to human health as they mediate key functions of metabolism and immunity (6, 12). It remains unclear whether the dysbiotic microbial communities associated with IBD are a cause or a consequence of the disease. However, both compositional changes and reduced microbial biodiversity are hallmarks of IBD and can even predict therapy failure in pediatric IBD (13). Alterations in gut microbial composition are often associated with more “proinflammatory” microbes, such as Proteobacteria phylum and *Ruminococcus gnavus* species, and decreased levels of butyrate-producing bacteria (e.g., *Faecalibacterium prausnitzii*), which correlated strongly in the presence of disease (10–12). Interestingly, dietary therapy is highly effective for pediatric CD, likely mediated by microbes, but is challenging to complete (14, 15). Increased incidence of IBD has been associated with a Western diet (16), and several studies have demonstrated the influence of diet on the gut microbiota (17–20). A number of fantastic reviews have been recently published (21, 22), illustrating the scientific evidence in support of the role that diet plays in the pathogenesis of IBD. Fibers have attracted specific attention and are generally considered beneficial to gut health. However, the reality is much more complex as some patients describe increased symptoms with fibers (23). Therefore, in the following review we will delve deeper, discussing the specific role of dietary fibers in health, and current evidence of fiber effects in IBD cell lines, animal models, and clinical trials, suggesting reasons why some IBD patients describe a sensitivity and worsening symptoms following fiber consumption.

## Dietary Fibers: Structures, Microbe Interactions, and Impacts on the Human Gut

**Figure 1:**
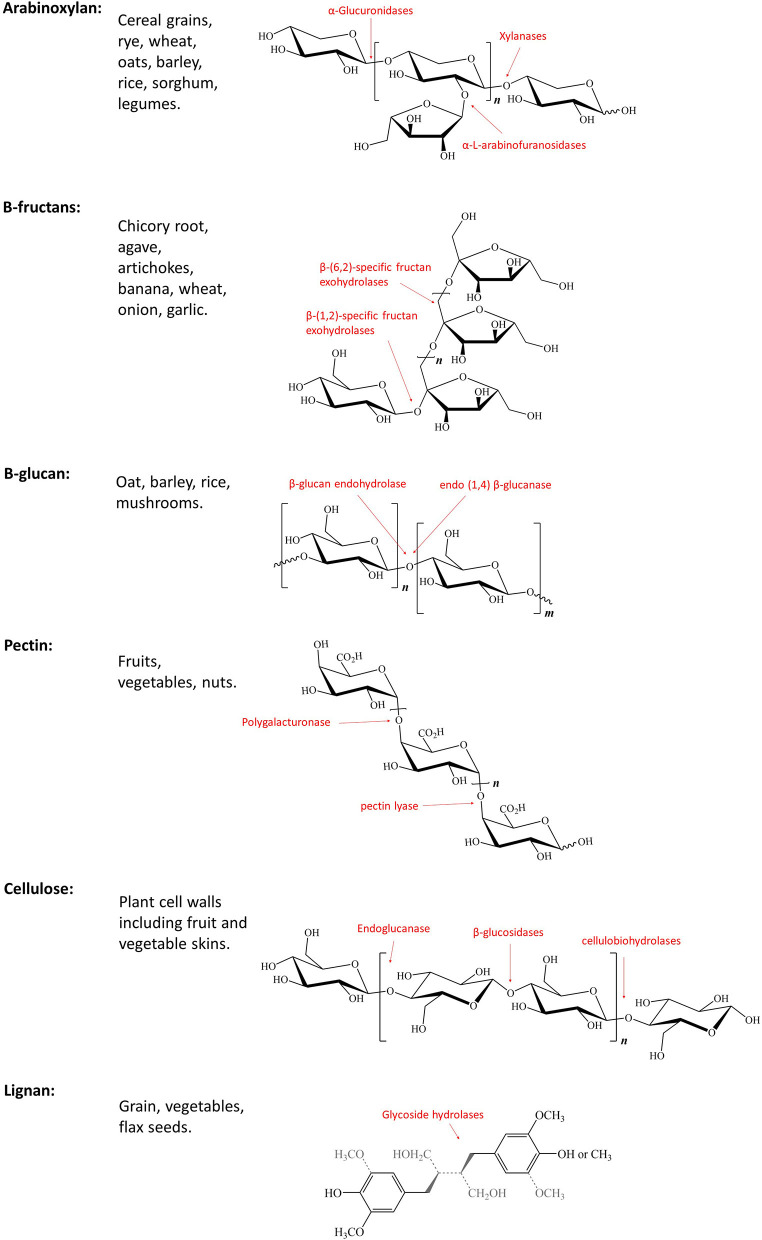
Not all Fibers are born equal; Structure, classification, and chemical features of key dietary fibers with key enzymes highlighted in red.

**Figure 2:**
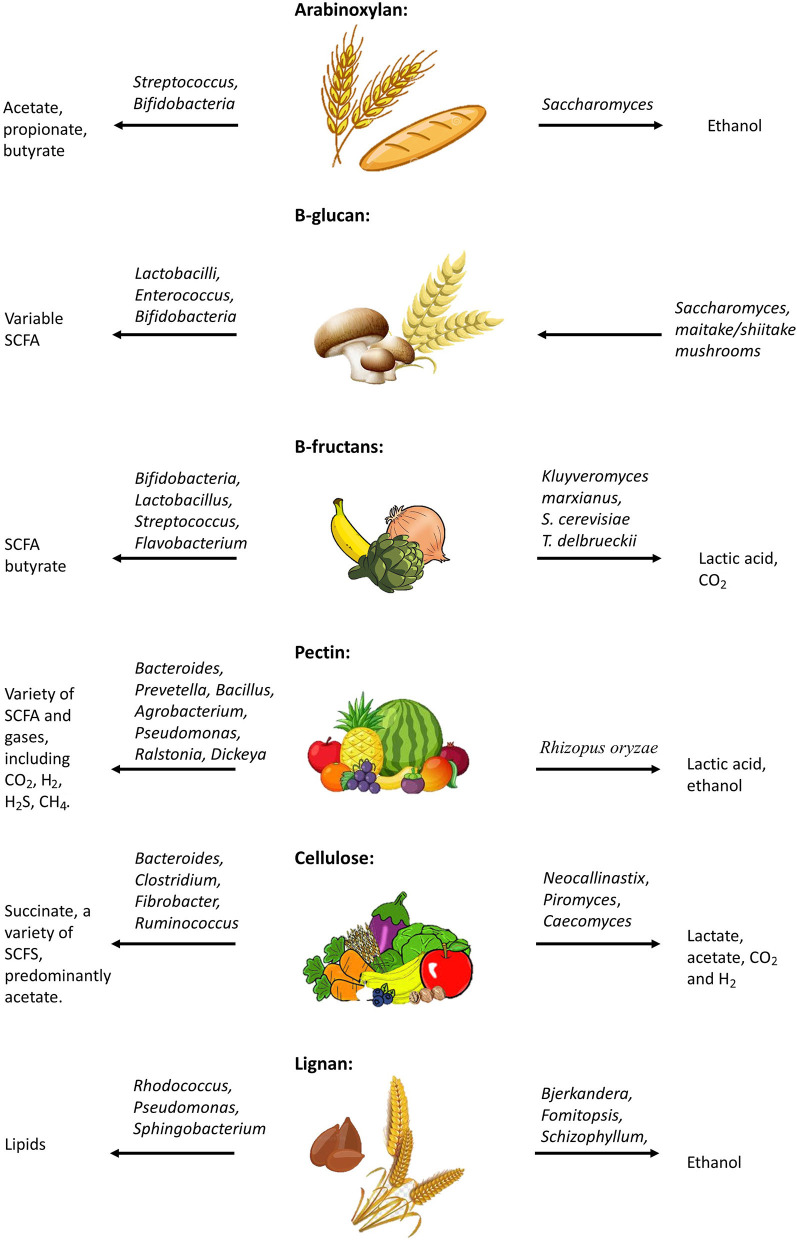
Metabolism of dietary fibers; Effects of dysbiosis on fermentation.

The term dietary fiber, first coined by Howeler, describes a complex group of non-digestible components of cell walls (Figure 1) (24). The term has grown to include non-starch polysaccharides (e.g., cellulose, pectin), non-carbohydrate-based polymers (e.g., lignan), resistant oligosaccharides (e.g., fructooligosaccharides, galatooligosaccharides), and carbohydrates considered to be of animal origin (e.g., chitin) (25). These dietary fibers can be found in a variety of food sources (Figure 2) and structurally differ in their chain length, linkage type, sugar components, and ability to associate with other chemical compounds (Figure 1) (25). Unlike most dietary components, non-digestible dietary carbohydrates (fiber and resistant starch) can withstand the acidity of the stomach and do not undergo degradation in the human small intestine (26); instead they are fermented by the gut microbiota consortium within the large bowel (Figure 2) where one microbe starts the fermentation and others continue the fermentation process, thereby working together systematically. This microbial consortium role thus highlights the potential implications of dysbiosis, which could alter or even prevent fiber fermentation. The colon houses one of the most complex (over 1,000 species) and highest populated microbiomes in the human body (70% of all microbes in the body) (27–29). Diet has been shown to be a key factor influencing the composition and functions of intestinal microbes, and continues to be examined as a tool in shaping the gut microbiota (30, 31). Studies suggest that while the recommended daily fiber intake from the Institute of Medicine (IOM) ranges from 19–38 g per day, dependent on age and gender (32), typical western diets lack fibers, and typically include roughly 13–20 g of dietary fiber per day, from which the commensal microbes are reliant upon for a source of energy and carbon (33–35). Most of the dietary fibers we consume originate from plant cell walls making up fruits, vegetables, and whole grains (25, 36). Dietary fibers consist of a variety of linked monosaccharides creating a variety of diverse molecules with varying side chains and physical variations, including physical arrangement and solubility (25). While these dietary fibers can be classified in a number of ways (37–40), the most common method of classification for nutritional purposes in humans segregates dietary fibers as water-soluble or insoluble (40). Water solubility within the gastrointestinal tract is related to the degree of fermentation by gut microbes (41). Soluble dietary fibers can increase digesta viscosity, which in turn delays gastric emptying and nutrient release, thus reducing glycaemic response (40, 42, 43). Examples of soluble dietary fibers include pectin, arabinoxylan, β-glucans, inulin, fructo-oligosaccharides, galacto-oligosaccharides, and xyloglucans (36). Insoluble dietary fibers, such as cellulose and lignin, on the other hand, are considered to be less valuable to the gut microbes as their strong hydrogen-binding networks reduce their accessible surface area to allow for fermentation (44). The complex structures of these dietary fibers require specific enzymes, such as carbohydrate-active enzymes (CAZymes) found only in microorganisms for degradation and utilization (25, 45). There are over 120 CAZenzymes identified to date, which have been shown to cleave glycosidic linkages (25). A number of key enzymes are highlighted in Figure 1, including those that target the breakdown of arabinoxylan (46), β-fructans (47, 48), cellulose (48), pectin (48), and lignin (49).

A variety of different microbes, that are able to utilize the same dietary fibers, coexist within the human bowel, resulting in a competitive environment that drives a variety of outcomes, based on the utility of these fibers (50). These selective differences in the microbiome of individuals results in distinct bacterial responses to consumption of dietary fibers (51, 52). Studies have demonstrated that this is likely because fermentation of dietary fibers is dependent on microbial community composition, wherein key microbes must be present for fermentation by their partner organisms to take place (52–55).

The human gut microbiome consists of at least 7 key phyla, predominated by Firmicutes (60–90%), Bacteroidetes (8–28%), Proteobacteria (0.1–8%), and Actinobacteria (2.5–5%) (56, 57). Studies suggest that a number of Bacteroidetes are involved in the breakdown of larger polysaccharides into smaller sugars, which can be utilized by Firmicutes that act as cross-feeders for other microbes (44). These microbes utilize a variety of complex pathways in the breakdown of dietary fibers to byproducts essential for human health. Fiber fermentation produces byproducts, such as short chain fatty acids (SCFAs) (58–63). Enzymes such as polysaccharidases, glycosidases, proteases, and peptidases are utilized to break complex carbohydrates or fibers into their sugar and amino acid components before fermenting these smaller components into SCFA, carboxylic acids, CO_2_, and H_2_ (64, 65).

The primary SCFAs produced by fiber fermentation include acetate, butyrate, and propionate, which have been generally shown to benefit the host through many mechanisms, including providing energy for colonic mucosa cells, reducing inflammation in IBD, and promoting differentiation and apoptosis of colonic cancer cells (66–68). A recent review by Williams *et al*. provides an excellent overview of the health benefits of these byproducts in humans (36). The precise mechanisms underlying the coordinated multi-microbe breakdown of dietary fibers into their byproducts and subsequent uptake within the gut continues to be illuminated in various healthy host settings. Changes in fermentation processes carry the potential to regulate changes in the gut microenvironment (60), immune modulation (69), and altered energy metabolism (70).

Interestingly, fiber-rich diets are associated with healthy growth of specific commensal microorganisms (71–76), many of which are directly responsible for SCFA production, improved gut mucosal barrier, and preventing inflammation. The amount and rate of SCFA production is dependent on the species and amounts of microbiota present within the gut (77), further illustrating how an altered gut microbiome in the presence of IBD impacts the production and absorption of protective SCFAs.

## Dietary Fibers and IBD: A Different Story?

Although there is consistent evidence to support the general concept that dietary fibers and their fermentation products are likely beneficial in the setting of IBD (78), it is important to recognize that much of this is an extrapolation of what we know from healthy state and that there are fundamental factors that would challenge this paradigm and potentially impact effects of fibers on IBD. First, fiber intake prior to developing IBD has been linked to risk of disease but having IBD has also been shown to result in changes within the patient's diet (79, 80). The same is true for microbes, which are obviously highly relevant to the impact of fiber on human health; they are thought to play a role in disease pathogenesis, but are clearly altered by having IBD (81). Surveys indicate that many patients (mostly with UC) tend to avoid consumption of dietary fibers (23). Some epidemiological studies, such as the EPIC-IBD cohort study, suggest there are no clear associations found to correlate development with IBD in relation to consumption of either total fiber or fiber from select sources (82). Other clinical studies suggest that long term intake of dietary fiber from fruits, and to a lesser degree, vegetables, reduces the risk of developing CD by up to 40%, with no effect on UC (83). These studies suggest that the protective effect of fiber for CD patients is mainly sourced from fruits and not associated at all with fiber intake sourced from whole grain or legumes (84–86). A restriction of dietary fiber consumption in “humanized” mice increases the consumption of colonic mucosa by colonic microbes, which has been suggested to contribute to reduced mucosa and inflammation in IBD (87). Many of the specific microbes affected by fiber-rich diets have been found to be less abundant in IBD (88–95), supporting the idea that fermentation of fibers may be altered in IBD patients. Additionally, animal studies have demonstrated that dietary fibers can inhibit IBD-associated inflammation (96–98), and clinical trials have shown that SCFA can prevent intestinal atrophy in IBD patients, allowing for tissue recovery (99).

## Fiber Therapies in IBD

Researchers continue to strive to understand the role that individual dietary fibers play in human health and in IBD patients to better appreciate how manipulating diet in these patients may help to improve clinical outcomes. Patients with ulcerative colitis showed a decrease in serum C-reactive protein (CRP) levels when given a fiber-based prebiotic, as well as a reduction in abdominal pain and cramping (100). A similar study by Fritsch *et al*. demonstrated a reduction of inflammatory markers and dysbiotic microbiome in patients with ulcerative colitis on a low-fat, high-fiber diet (101). Similarly, mice given a probiotic and fiber-based prebiotic displayed amelioration in disease activity and histological score compared to a prebiotic alone, and a reduction in serum CRP levels (102). Recent studies have shown that Institute of Cancer Research (ICR) mice fed a fiber-free diet displayed shortening of colon length, which was found to be an indicator for IBD, and reduced microbial diversity (103). This indicates that dietary fibers play an important role in mitigating disease severity in IBD patients.

Current studies further support the SCFA butyrate as an anti-inflammatory product of fermentation within the colon that may improve epithelial barrier integrity (104–107). However, it is important to also highlight that there remains contradictory evidence from studies that have shown that butyrate had no benefit in these patients (108, 109). Unfortunately, many clinicians continue to promote reduced fiber consumption, or fiber avoidance altogether, in IBD patients (particularly UC) while there is no evidence to support this as a relevant therapeutic intervention (110, 111). While it remains unclear exactly which factors modulate the health benefits associated with dietary fibers in IBD, results from studies performed to date suggest that modulating diet through prebiotic/probiotic therapies to promote select fiber fermentative microbes may aid in improving IBD patient symptoms.

## This Section Reviews Specific Fibers and Their Potential Role in the Setting of IBD

Key features of the following dietary fibers are summarized in Table 1.

**Table 1:** Summary of sources, fermentation, products, and potential impacts of dietary fibers.

**Fiber**	**Common diet Source**	**Fermenting microbes**	**Products of Fermentation**	**Impact on Host**
Arabinoxylan	Cereal grains, rye, wheat, oats, barley, rice, sorghum, legumes	*Streptococcus, Bifidobacteria*	Acetate, propionate, butyrate	Anti-inflammatory in mice via IFN-γ Th cells. Anti-inflammatory in cell lines via COX-2. Reduced mucin-degrading microbes
		*Saccharomyces*	Ethanol	
β-glucan	Oat, barley, rice, mushrooms	*Lactobacilli, Enterococcus, Bifidobacteria*	Variable amounts of SCFA depending on source and microbes present	Pro-inflammatory response to fungal cell wall β-glucan. Reduced IBD symptoms in DSS mice
β-fructans	Chicory root, agave, artichokes, banana, wheat, onion, garlic	*Bifidobacteria, Lactobacillus, Streptococcus, Flavobacterium*	SCFA, primarily butyrate	As a prebiotic they reduced intestinal inflammation in mice. Reduced IBD symptoms in DSS mice
Pectin	Fruits, vegetables, nuts	*Bacteroides, Prevetella, Bacillus, Agrobacterium, Pseudomonas, Ralstonia, Dickeya*	Variety of SCFA and gases, including CO_2_, H_2_, H_2_S, CH_4_	Directly inhibits pro-inflammatory cytokines in mice. *In vitro* studies showed reduce pro-inflammatory cytokines
Cellulose	Plant cell walls including fruit and vegetable skins	*Bacteroides, Clostridium, Fibrobacter, Ruminococcus*	Succinate; a variety of SCFA, predominantly acetate	Shifts gut microbiome in mice. Protective against colitis and anti-inflammatory in mice
Lignan	Grain, vegetables, flax seeds	*Bjerkandera, Fomitopsis, Schizophyllum*	Ethanol	Improved lipid abnormalities and reduced systemic inflammation in mice
		*Rhodococcus, Pseudomonas, Sphingobacterium*	Lipids	

*Detailed information available in the text*.

We will first present *soluble fibers*, followed by insoluble.

### Arabinoxylan

Arabinoxylan (AX) is a hemicellulose molecule composed of a β-(1,4)-linked xylose backbone containing arabinose side chains (112, 113). The detailed structure of AX is dependent on several factors, such as its source, enzymes used for hydrolysis, and methods of extraction (114). AX is a large component of dietary fiber found in cereal grains and other plant and animal food sources (115–117). The fermentation of AX by gut microbes such as *Streptococcus* and *Bifidobacteria* results in the production of SCFAs, including acetate, propionate, and butyrate (115, 118–121). Furthermore, downstream fungal microbes, such as *Saccharomyces cerevisiae* result in fermentation processes producing biomass and ethanol (122).

Due to the previously stated structural differences that can exist within AX molecules, it is important to consider the polymer side branching and source of AX fiber when discussing immunomodulatory properties of this fiber (123–125). Studies have shown that oral administration of corn-husk AX fiber has anti-inflammatory effects in mice, through the activation of an INF-γ dependent T helper 1-like immune response (126). AX can also stimulate COX-2 through TLR-4 upregulation, as well as contributing to a reduction in pro-inflammatory cytokines IL-8 and TNF-α in colon cancer cell lines (127). Prebiotic administration of long-chain AX resulted in decreased abundance of mucin-degrading microorganisms and led to a three-fold increase of cecal mucin levels in rats (128). This is especially important in the context of IBD as the protective mucin layer within the gut that aids in providing a healthy barrier against environmental threats, is greatly diminished in IBD patients (129).

An AX enriched food, germinated barley (GB), was used in a clinical trial, in which 59 UC patients in remission were divided into two groups; 37 individuals in the control group received a conventional drug for 1 year and the other 22 patients in the GB group received a conventional drug plus 20 g of GB daily. GB significantly ameliorated the disease activity index and reduced the recurrence rate compared to control group, and no significant side effects were observed (130). Neyrinck *et al*. reported that the abundance of *Bacteroides* and *Roseburia spp*., as well as *Bifidobacteria*, were increased with wheat AX supplementation, and that the gut barrier function was strengthened while serum pro-inflammatory markers were reduced (131). In another 8-week clinical trial, 19 UC patients with wheat bran AXs and resistant starch treatments were shown to shape the microbial community composition, which increased diversity within the *Clostridium cluster XIVa* compared to the control group (132). *Clostridium cluster XIVa* and *Bifidobacteria* have gained much attention recently due to their contribution to gut homeostasis, by preserving gut barrier functions and exerting immunomodulatory and anti-inflammatory properties (133).

### β-glucan

β-glucans are a family of naturally occurring glucose polysaccharides that make up the cell wall of select bacteria and fungi, but are also found in dietary plant cells, such as oat and barley (134, 135). Interestingly, studies focused on the immune response to fungal infection indicate that β-glucan on the surface of fungal cells interacts with host immune cell receptors (*e.g*., Dectin-1), inducing a pro-inflammatory response (47–49). Dietary β-glucans differ from those found in fungal cell walls by level of solubility and downstream effects (136, 137). Dietary β-glucans have been shown to be fermented by microbes such as *Lactobacilli, Enterococcus*, and *Bifidobacteria*, forming a variable amount of SCFAs, depending on the source of fiber along with the microbial species involved (138, 139). Furthermore, production of the β-glucan receptor, Dectin-1, has been shown to be increased in dextran sodium sulfate (DSS)-induced colitis model mice (140). Inhibition of Dectin-1 has been shown to ameliorate colitis in this model (141). The DSS mouse model was recently utilized to examine inflammatory responses to a series of different glucan preparations (142). Findings from this study suggest that histology disease scores were reduced in response to fungal extracted glucans, which were associated with Dectin-1. Specifically, the glucans most effectively associated with reduction of IBD-like symptoms in DSS mice were those collected from the edible mushroom *Pleurotus eryngii* (142). Meanwhile other studies have demonstrated that oral administration of various other isolates of β-glucan in fact worsened intestinal inflammation in DSS-induced colitis models (143). These results highlight the importance of understanding the chemical structures of different dietary fibers and their direct interactions within the bowel to potentially aid in developing dietary recommendations to promote improved gut health in IBD patients.

### *B*-Fructans

The β-(2 → 1) linked fructose oligo- and poly-saccharides known as β-fructans (inulin and oligofructose/FOS, respectively), are commonly found in plant sources including chicory root, agave, and artichokes, while other sources (banana, wheat, onion, garlic) contain lesser amounts (144). Microbes such as *Bifidobacteria, Lactobacillus, Streptococcus, Flavobacterium*, and a variety of baking yeast have been shown to be responsible for fermentation of β-fructans (144, 145). Butyrate-producing bacteria have primarily been demonstrated to be positively affected by these fibers, resulting in a wide variety of health benefits (146–149). As a prebiotic, these fibers have demonstrated benefits for the treatment of intestinal inflammation in mouse models of IBD, as described in a recent series of review articles (150–153). Overall, DSS-induced IBD-like symptoms were reduced in mice fed with β-fructans, or relapse was prevented, through the mediation of antioxidative defense mechanisms, identified in more recent studies in animal models and cell lines (154, 155). β-fructans have also been shown to interact with carbohydrate receptors (GLP-1R), affecting reactive oxygen species (ROS) production and associated inflammation (144, 156–158). Interestingly, while inulin has been demonstrated to have positive effects on inflammation in select situations, a number of studies have also suggested inulin can exacerbate the severity of colitis in an IL10^−/−^ and DSS-model of colitis (159), and promote HCC-progression in mice (160).

### Galactooligosaccharides

Galactooligosaccharides (GOS) are oligosaccharides composed of different galactosyl residues (from 2 to 9 units) and terminal glucose molecules linked by β-glycosidic bonds (161). They naturally are found at low concentrations in the milk of many animals, including humans and cows, but they also can be produced by chemical glycosylation or biocatalysis of lactose (162). In a human randomized, double-blind, placebo-controlled study, the effect of 6-weeks daily intake of 5 g GOS on intestinal barrier function and gut microbiome composition was evaluated in 114 obese individuals. The GOS showed a bifidogenic effect on the resident gut microbiota, and improvement on colonic permeability (163). *Bifidobacteria* have gained emerging attention recently because of their contribution to gut homeostasis, by preserving gut barrier functions and exerting immunomodulatory and anti-inflammatory properties (133). Thus, *Bifidobacteria* are generally considered to be health-promoting organisms and constitute one of the main groups of organisms targeted by prebiotics. A human study reported the positive effects of GOS with daily dosage 5 or 10 g on *Bifidobacterium* strains from these genera have been elucidated, while a lower dose of 2.5 g showed no significant effect (164). GOS has also been demonstrated to enhance *F. prausnitzii* levels (165). *F. prausnitzii* is a commensal bacterium with suggested anti-inflammatory effects, supported by gut microbiota analysis of CD patients and functional studies (166). A decrease of *F. prausnitzii* abundance in the gut is typically recognized as a signature of gut dysbiosis in CD, compared to healthy people (167). Furthermore, 44 patients with irritable bowel syndrome (IBS) were randomized to receive 3.5 g/day GOS, 7 g/day GOS, or 7 g/day placebo over 12 weeks. Results showed that GOS acted as a prebiotic via stimulating gut *Bifidobacteria* in IBS patients, and effectively alleviated IBS symptoms (168). Given that several important symptoms, including pain and diarrhea, overlap in IBD and IBS, these interventions may also provide references to use prebiotics for IBD treatment or adjuvant treatment, but this needs to be proven in studies. Together, recent evidence suggests that the prebiotic effects of GOS on the specific gut microbiota, such as *Bifidobacterial* consortia and *F. prausnitzii* with the resulting oligosaccharide degradation, might offer a potential mechanism to improve gut barrier function, and suppress inflammation.

### Pectin

Pectin is a complex polysaccharide found in the cell wall of fruits and vegetables (169). It is made up mostly of α-1,4-linked D-galacturonic acid residues with variable levels of esterification between plant species that results in different effects on fermentation, SCFA production, and effects on the immune profile (170, 171). A variety of microorganisms (*e.g., Bacteroides, Prevetella, Bacillus, Agrobacterium, Pseudomonas, Ralstonia, Dickeya, and yeast*) utilize enzymatic processes (*e.g*., isomerase) or oxidative pathways, in the almost complete fermentation of pectin within the colon, resulting in the production of a wide variety of SCFAs along with a number of gases, including CO_2_, H_2_, H_2_S, and CH_4_ (169, 172–177). Pectin directly inhibits the pro-inflammatory cytokine toll-like receptor (TLR)1&2 pathways and prevents ileitis in mice (53). *In vitro* studies have shown that pectin reduces the pro-inflammatory cytokine IL-1β and interacts with and down regulates TLR-4 signaling (178). Dietary pectin also moderates the production of pro-inflammatory cytokines and immunoglobulins, working to downregulate inflammatory response in the colon of mice (179). A recent study examining the variable effects of pectin side chain content on colitis induced C57BL/6 mice demonstrated that a diet high in pectin (orange pectin) ameliorated disease compared to low pectin (citrus pectin) or no-pectin diet supplementation (180).

### Insoluble Fibers

#### Cellulose

Cellulose is the main structural component of plant cell walls and is composed of linear chains of β(1 → 4) linked glucose monomers (181, 182). While insoluble fibers are considered to be much less important for the production of healthy byproducts of fermentation due to the limitations in breaking down these components, microbes (*e.g., Bacteroides, Clostridium, Fibrobacter, Ruminococcus*, and anaerobic fungi) have been demonstrated to utilize cellulose for the production of intermediate byproducts of fermentation, such as succinate, along with a variety of SCFA byproducts, predominantly acetate (149, 183–185). This means that degradation of cellulose provides the opportunity for a variety of responses within the bowel; however, in the context of IBD it is important to highlight that the byproduct succinate has been associated with inflammation, suggesting one reason that may help explain why some patients experience sensitivity to dietary fibers (186, 187). Mice fed a high-cellulose diet were shown to have increased expression of *Mt1* and *Mt2* genes (188, 189), which have been found to play a protective role in inflammation (188, 190) and a colitis model through anti-apoptotic and immune-modulating effects (188, 191). Experimental studies in mice have demonstrated that dietary supplementation of cellulose contributes to substantial shifts in the gut microbiome, which were associated with transient trophic and anticolitic effects (192). A recent study has indicated that a high-cellulose diet maintains gut homeostasis and ameliorates gut inflammation by altering gut microbiota and metabolites in mice, and can be protective against colitis (188). In this study, low-cellulose diets were found to cause crypt atrophy, goblet cell depletion, and upregulation of pro-inflammatory genes in the colon (188). Additionally, dietary cellulose is thought to be associated with increased abundance of *Akkermansia*, which promote gut barrier function, and enhanced mucin secretion by goblet cells (188). Interestingly, cellulose has been found to improve survival in the murine model of sepsis through systemic anti-inflammatory effects. High-fiber cellulose diets were associated with a decrease in serum concentration of pro-inflammatory cytokines, reduced infiltration of neutrophils in the lungs, and decreased hepatic inflammation (193).

#### Lignan

Lignan is a complex polymer containing ~40 oxygenated phenylpropane units that have undergone a dehydrogenative polymerization process and is a non-carbohydrate component of cell walls (182). Lignan can be found in many plants including grains and vegetables, and the highest concentration is found in flaxseed (194). Much like cellulose, the breakdown of lignan is complex and difficult to achieve. Certain wood-degrading fungi (*e.g., Bjerkandera, Fomitopsis*, and *Schizophyllum*) have been shown to utilize peroxidase, ligninase, and laccase enzymes in the degradation of lignin, producing ethanol (Figure 1) (195–197). Interestingly *Rhodococcus, Pseudomonas*, and *Sphingobacterium* are able to use the aromatic components of lignan during its breakdown process in order to produce high levels of lipids for biofuel production (198–201). These data suggest that there may also be some gut microbiota capable of some level of lignin fermentation processes. Lignan was found to contribute to decreased white blood cell counts and proinflammatory/profibrogenic cytokine levels, as well as decreased gene expression of cytokines and cytokine receptors in mice exposed to asbestos (202). A study conducted on hemodialysis patients showed that supplementation of flaxseed in the diet contributed to improved lipid abnormalities and reduced systemic inflammation (203).

These studies highlight the importance of studying the variability in response to specific dietary fibers (or similar molecular structures), while most studies continue to evaluate fiber diets in a holistic manner, lumping all dietary fibers into one.

## Recommendations for IBD Patients Based on Current Evidence

Based on the studies published to date, there remains clear evidence for the importance of high intake of dietary fibers overall with no current evidence to support restriction of dietary fibers in patients without intestinal strictures or obstructions (21). However, the benefits of the specific types and sources of dietary fibers remain poorly understood, and the research paradigm has begun to shift toward examining the direct effects of *individual fiber types* in IBD. As many patients describe a sensitivity to dietary fibers (23), it seems wise to recommend that the increased source of dietary fiber come from those fibers with clear evidence to support anti-inflammatory or preventative effects as speculation remains surrounding the negative impact of certain fibers on patient health during IBD flares (110). A recent review of current literature by Levine and colleagues suggests that there is an acceptable amount of data to support recommended moderate to high amounts of fruit and vegetables in CD patient diets, while there remains insufficient evidence to support these recommendations in UC patients (21). Notably, the evidence we have discussed in the previous sections suggests that gut microbes more readily ferment soluble dietary fibers, and the SCFA, butyrate, seems to be the most beneficial byproduct studied to date, providing a source of carbon and energy for the colon epithelium (204). It could be said that specifically those fibers utilized for the production of increased butyrate should be included in greater quantities in the diet of IBD patients, while due to a lack a consistent evidence to support a beneficial effect in IBD patients, insoluble fiber consumption should perhaps be avoided or reduced, especially for those patients with active disease (21). Specifically, arabinoxylans, β-glucans, β-fructans, and pectins, which are all found in vegetables, fruits, and grains, have been shown to be valuable sources of SCFAs by butyrate-producing microbes (21). So what is the easiest means of increasing consumption of these dietary fibers while limiting insoluble fiber consumption? Food products such as bananas and broccolis, nuts, tomatoes, and carrots, mushrooms, and peeled apples and citrus fruits contain high levels of soluble dietary fibers while limiting insoluble fiber intake (205). It is important to note that the evidence in support of this includes very limited numbers of human studies, and a number of contradictory animal and *in vitro* studies, however, these recommendations may help certain individuals. Interindividual variability in microbial composition and function will also need to be considered, as further discussed below (118).

### The Pediatric Angle to the Fiber Story

Some of these considerations require additional attention in the pediatric setting. First, the first-line therapy for pediatric-onset CD, as recommended by the revised ECCO-ESPGHAN guidelines, exclusive enteral nutrition (EEN) (206), is based on liquid diet that does not include fiber in most cases. Would selective addition of soluble fiber to these controlled diets further improve their effect? The Crohn disease exclusion diet (CDED) has utilized some of the principles discussed in this review in the design of the food-based diet (19). CDED has shown at least equal success to EEN, but even better tolerance, which is also coupled by some of the expected changes in microbial composition (20, 207). However, as many of the origins of IBD pathogenesis are likely related to early life events, including early commensal engraftment of the infant gut, it is possible that what we feed our microbes (*i.e*., fibers) may have an impact on developmental immune responses related to many chronic conditions, including IBD. This might explain the beneficial effect of breastfeeding (through human milk oligosaccharides, such as GOS), as well as other links to diet (208). Once the science linking diet through microbes to chronic disease is further developed, we would expect that better guidance on early life fiber exposure could help prevent IBD at a later age.

## Conclusions

With an increasing focus on nutritional interventions, especially in children with Crohn disease, and the interest (but still limited supportive research) on specific use of probiotics and prebiotics in IBD, it is important that we broaden our understanding of how foods affect the bowel, especially in regards to the fiber fermentation processes that occur in the bowel. Results from hundreds of studies to date have demonstrated the ability of select individual microbes or whole microbe cultures, in rats, mice, rabbits, swine, poultry, and even select studies in humans, to manipulate fermentation of dietary fibers (209–213). But how do these *in vitro* and *in vivo* studies translate to the complex system of the human bowel? As this review highlighted, differences in microbial composition and dietary factors present can result in substantial differences in host inflammatory response (21). Taking into account that each select microorganism utilizes a variety of different environmental sources from the host diet and microenvironment for carbon and energy, along with the dysbiotic nature of the microbiome of IBD patients, there is a clear possibility that incomplete fermentation, potentially due to dysbiotic microbiome, can result in buildup of pro-inflammatory byproducts such as succinate (214). Evidence also suggests an alternative option; specifically, when macrophage cells are stimulated by lipopolysaccharide (LPS), such as that found on pathobionts, a metabolic shift occurs, resulting in accumulation of succinate (214, 215). This in turn promotes biological pathways responsible for the production of pro-inflammatory cytokines, such as interleukin (IL) 1β, suggesting possible avenues for future therapeutics (66, 216). However, with the right combination of microbes and environmental factors, each of these fiber subtypes have been shown to provide anti-inflammatory benefits as highlighted above. It is important to re-highlight here that the gut microbes work in consort and therefore, it is not just one microbe species performing these fermentative processes in isolation; this adds to the complexity of the gut environment.

### Research Gaps and Future Directions

The precise interaction of whole chain fibers with the human bowel remains under-studied and complete answers to many questions still remain. How does the dysbiotic nature of IBD affect fiber fermentation? And how does this relate to the sensitivity that patients experience to dietary fibers, especially during disease flare ups? While sequencing and culture techniques continue to improve our ability to examine these system interactions, there remains a gap in understanding how these factors all align when it comes to microbes, nutrition, and gut health. Considering it is the gut microbiome that is responsible for the fermentation of dietary fibers, and that there is a great deal of variability among individuals, let alone IBD patients, one of the keys to understanding the role of dietary fibers in IBD, is likely in understanding which microbes must be present and/or absent for the healthy fermentation and production of SCFA to occur (18, 213, 217–219). This idea highlights the importance of patient-specific medicine. In this instance, future therapies will likely begin with profiling the gut microbiota of individuals and identifying a specific combination of dietary recommendations, prebiotic, and probiotic therapies, directed toward re-establishing a healthy, balanced microbiome capable of providing the appropriate sources of carbon and energy via fermentation. We believe that it is imperative to advance our understanding of fiber fermentation within the IBD gut to aid in improving dietary guidelines and therapeutic options, in order to promote the growth of a healthy microbiota and increase beneficial SCFAs in patients.

## Author Contributions

HA structured the manuscripts draft. HA and DA constructed all figures. All authors contributed to writing and editing the manuscript.

## Conflict of Interest

The authors declare that the research was conducted in the absence of any commercial or financial relationships that could be construed as a potential conflict of interest.
